# Correction for David et al., “Inactivation mechanisms of influenza A virus under pH conditions encountered in aerosol particles as revealed by whole-virus HDX-MS”

**DOI:** 10.1128/msphere.00595-24

**Published:** 2024-09-19

**Authors:** Shannon C. David, Oscar Vadas, Irina Glas, Aline Schaub, Beiping Luo, Giovanni D'angelo, Jonathan Paz Montoya, Nir Bluvshtein, Walter Hugentobler, Liviana K. Klein, Ghislain Motos, Marie Pohl, Kalliopi Violaki, Athanasios Nenes, Ulrich K. Krieger, Silke Stertz, Thomas Peter, Tamar Kohn

## AUTHOR CORRECTION

Volume 8, no. 5, e00226-23, 2023, https://doi.org/10.1128/msphere.00226-23. Due to a labeling error upon receipt of the influenza A virus strain, this study was conducted under the assumption that it was A/WSN/33. During recent sequencing in an unrelated project, it has come to light that the strain used in this study was in fact A/Puerto Rico/8/34 (typically abbreviated A/PR8). This correction to the strain does not change any of the outcomes or findings of the study, as A/PR8 and A/WSN/33 are both highly lab-adapted isolates of influenza A virus subtype H1N1, both with spherical virion morphology, >90% consensus in their HA protein sequences, and >96% consensus in their NP and M1 protein sequences. They also have an identical fusion pH of 5.1 (see original reference 81). The A/PR8 strain was provided by Dr. Jovan Pavlovic at the Institute of Medical Virology, UZH. The recombinant VSV strains used in this work remain unchanged.

The sequence analyses and HDX-MS results also remain unaffected and unchanged; in fact, the A/PR8 HA sequence was already used for analysis in the paper. Figures S8 and S9 have been updated to reflect the A/PR8 HA sequence (accession number P03452) rather than the A/WSN/33 HA sequence (accession number I4EPC4), with no change in outcome nor any required changes to the text or cited homology percentages compared to the other strains. The correct figures are given in the revised supplemental file in this correction.

## SUPPLEMENTAL MATERIAL

Revised Supplemental Material msphere.00595-24-s0001.pdf Revised version of the Supplemental Material file containing Figures S1 to S9 and Tables S1 and S2.

ASM does not own the copyrights to Supplemental Material that may be linked to, or accessed through, an article. The authors have granted ASM a non-exclusive, world-wide license to publish the Supplemental Material files. Please contact the corresponding author directly for reuse.

