# Supplementary material for: Correction for David et al., “Inactivation mechanisms of influenza A virus under pH conditions encountered in aerosol particles as revealed by whole-virus HDX-MS”
Source: mSphere. 2024 Sep 19;9(10):e00595-24. doi: 10.1128/msphere.00595-24 (PMC11520293; doi:10.1128/msphere.00595-24)
Supplement: Revised Supplemental Material — Revised version of the Supplemental Material file containing Figures S1 to S9 and Tables S1 and S2. [file msphere.00595-24-s0001.pdf]

SUPPLEMENTARY INFORMATION for:

Inactivation mechanisms of Influenza A virus under pH conditions encountered in aerosol particles as revealed by whole-virus HDX-MS.

Shannon C. David[a], Oscar Vadas[b]*, Irina Glas[c]*, Aline Schaub[a], Beiping Luo[d], Giovanni D'Angelo[e], Jonathan Paz Montoya[e], Nir Bluvshtein[d], Walter Hugentobler[f], Liviana K. Klein[d], Ghislain Motos[f], Marie Pohl[c], Kalliopi Violaki[f], Athanasios Nenes[f,g], Ulrich K. Krieger[d], Silke Stertz[c], Thomas Peter[d], and Tamar Kohn[a,#].

[a] Environmental Chemistry Laboratory, School of Architecture, Civil and Environmental Engineering, Ecole Polytechnique Fédérale de Lausanne (EPFL), Lausanne, Switzerland

[b] Protein Platform, Faculty of Medicine, University of Geneva, Geneva, Switzerland

[c] Institute of Medical Virology, University of Zurich, Zürich, Switzerland

[d] Institute for Atmospheric and Climate Science, ETH Zurich, Zurich, Switzerland

[e] Laboratory of Lipid Cell Biology, Interschool Institute of Bioengineering and Global Health Institute, School of Life Sciences, Ecole Polytechnique Fédérale de Lausanne (EPFL), Lausanne, Switzerland

[f] Laboratory of Atmospheric Processes and their Impacts, School of Architecture, Civil and Environmental Engineering, Ecole Polytechnique Fédérale de Lausanne (EPFL), Lausanne, Switzerland

[g] Institute of Chemical Engineering Sciences, Foundation for Research and Technology Hellas, Patras, Greece

* Equal contribution

# Corresponding author: tamar.kohn@epfl.ch

Running title: Whole-virus HDX-MS of Influenza A virus

Key words: Aerosols / HDX-MS / Influenza virus / Matrix 1 / Haemagglutinin

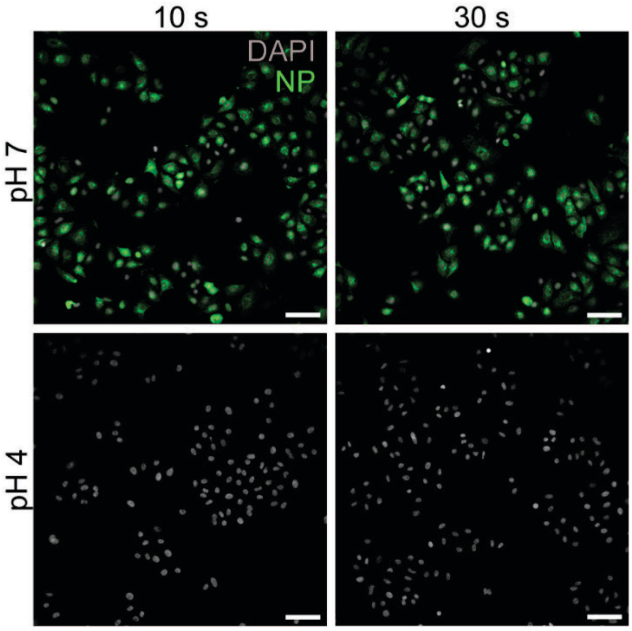

Supplementary Figure S1 – Immunofluorescence images of A549 cells infected with IAV samples treated at pH 4 or 7 for 10 or 30 seconds. All samples were neutralized prior to cell infections. A549 cells were infected at a multiplicity of infection (MOI) of 2, and IAV protein production was visualized by immunofluorescent staining of the nucleoprotein (NP) at 6 hours post-infection. Scale bar corresponds to 250 µm. Images are representative of duplicate experiments.

Haemagglutinin (HA)
P03452

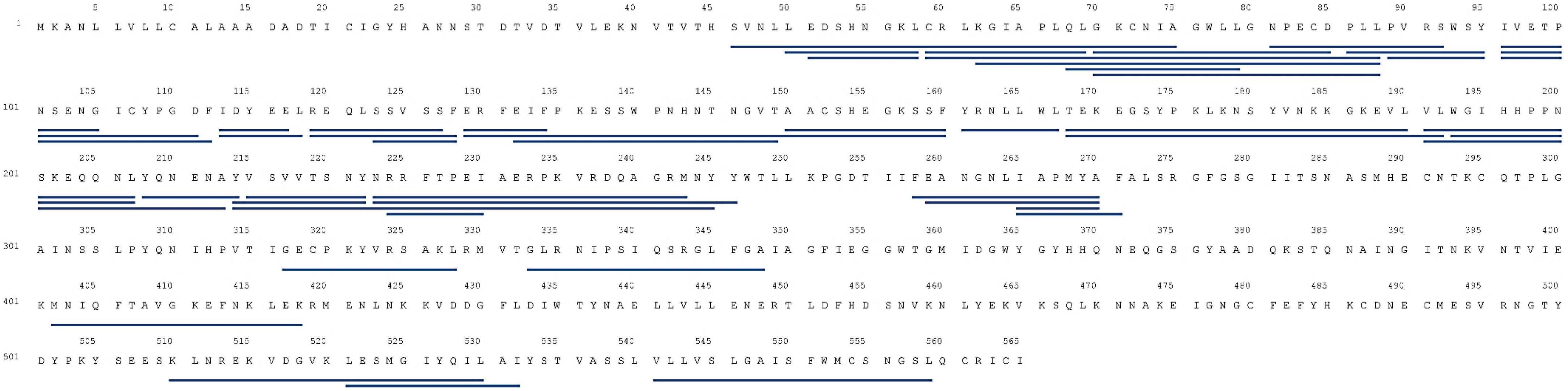

Supplementary Figure S2 – HDX-MS peptide coverage map displaying unique peptides for influenza A virus HA. Whole virus samples were acidified to pH 4, then neutralized back to pH 7 after 30 seconds. Samples were then incubated in D_2O, quenched and processed for analysis by HDX-MS. Peptides aligned to published HA protein sequence for strain A/PR8 (UniProt: P03452). Only unique peptides where HDX-MS data was attained for acid-treated samples are shown here. Under these conditions, 74% of the HA1 protein subunit (residues 18 – 343) and 30% of the HA2 protein subunit (residues 344 – 565) were covered by at least 1 unique peptide. Coverage of HA2 was limited compared with HA1, which is attributed to protease resistance and steric occlusion from high HA2 density on intact IAV virions. Coverage maps made using HDExaminer (Sierra Analytics).

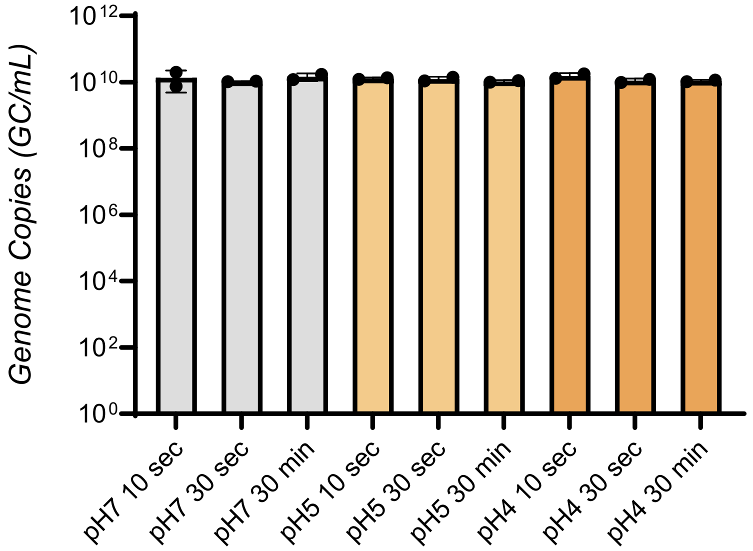

Supplementary Figure S3 – Pre-treatment with acidic pH does not alter amplifiable copies of viral RNA, even after 30 minutes. RT-qPCR results showing the IAV genome region tested (M2 genome segment) is able to be replicated to comparable levels regardless of pH 4, 5 or 7 pre-treatments for 10 seconds, 30 seconds, or 30 minutes at room temperature. Samples were all neutralized back to pH 7 prior to RNA extraction and quantification by RT-qPCR. Data presented as mean genome copies (GC/mL) ± SD for duplicate samples.

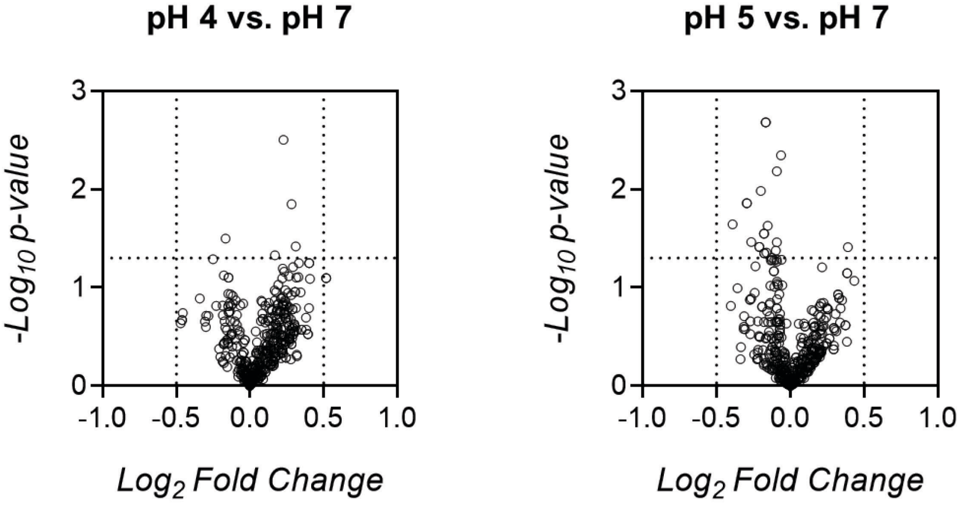

Supplementary Figure S4 – Pre-treatment with acidic pH does not significantly alter lipid classes of the IAV envelope. Volcano plots obtained by comparing the relative levels of lipid species in samples exposed to pH 4 (left panel) or pH 5 (right panel) to samples exposed to pH 7. N = 3 samples per treatment group, each data point indicates an individual lipid species, vertical dotted lines are drawn to highlight size effects > 0.5 or < -0.5 Log_2-Fold change, horizontal dotted lines are drawn to highlight significant changes ($-Log_{10}$ p-values > 1.301; i.e., p-value < 0.05).

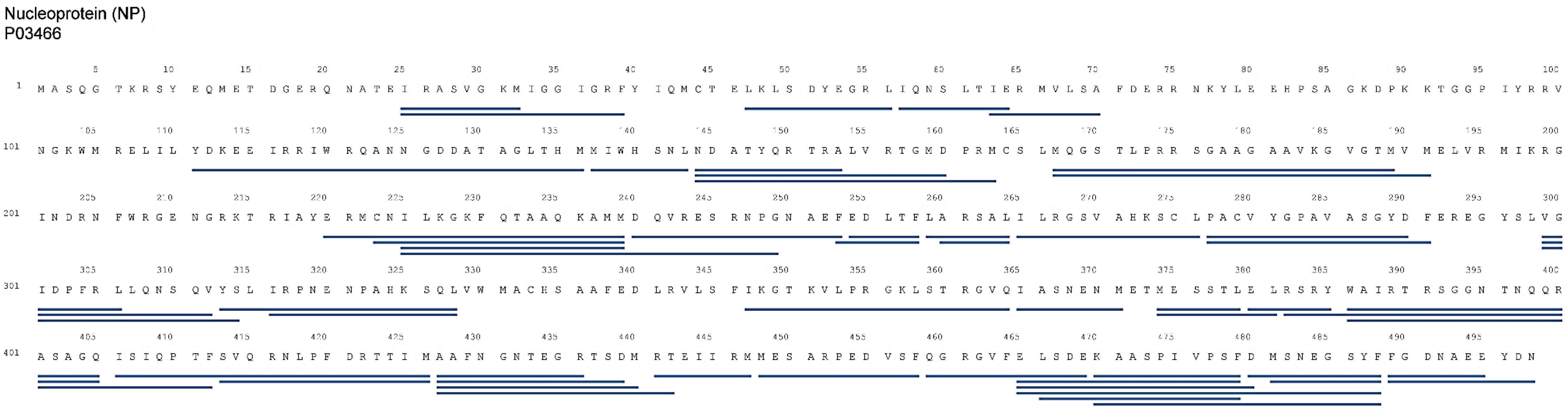

Supplementary Figure S5 – HDX-MS peptide coverage map displaying unique peptides for influenza A virus NP. Whole virus samples were acidified to pH 4, then neutralized back to pH 7 after 30 seconds. Samples were then incubated in D_2O, quenched and processed for analysis by HDX-MS. Peptides aligned to published NP protein sequence for strain A/WSN/33 (UniProt: P03466). Only unique peptides where HDX-MS data was attained for acid-treated samples are shown here. In these conditions, 75% of the NP protein was covered by at least 1 unique peptide. Coverage was evenly distributed across the NP protein, with slightly more unique peptides appearing at the C-terminal end. Coverage maps made using HDExaminer (Sierra Analytics).

Matrix Protein 1 (M1)
P05777

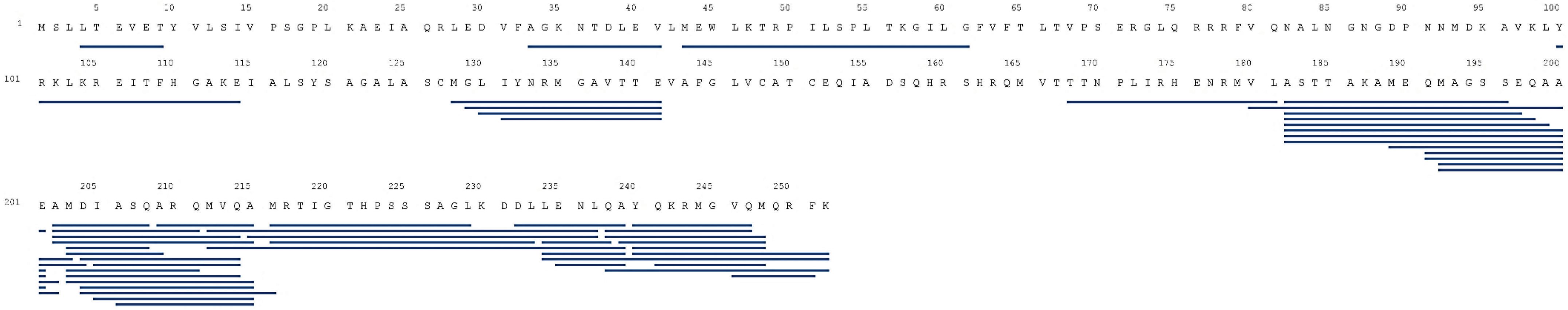

Supplementary Figure S6 – HDX-MS peptide coverage map displaying unique peptides for influenza A virus M1. Whole virus samples were acidified to pH 4, then neutralized back to pH 7 after 30 seconds. Samples were then incubated in D_2O, quenched and processed for analysis by HDX-MS. Peptides aligned to published M1 protein sequence for strain A/WSN/33 (UniProt: P05777). Only unique peptides where HDX-MS data was attained for acid-treated samples are shown here. In these conditions, 68% of the M1 protein was covered by at least 1 unique peptide. Coverage of the N-terminal region was limited compared with the C-terminal, which is attributed to proximity of the N-terminal region to the viral envelope and potential protease inhibition. Coverage maps made using HDExaminer (Sierra Analytics).

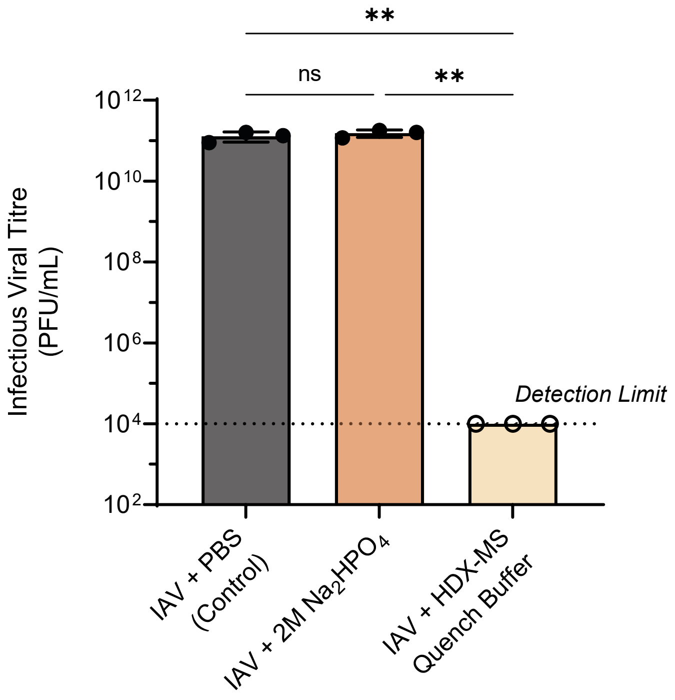

Supplementary Figure S7 – IAV infectious titer is not impacted by pH neutralization method. Purified A/WSN/33 IAV stock (7.5 x 10^{11} PFU/mL) was mixed with 2M Na_2HPO_4 to mimic the pH neutralization process, in triplicate. Samples were vortexed to mix, then diluted 1/100 in PBSi for freezing, prior to titration by plaque assay. Control virus was mixed with an equivalent volume of PBS in the absence of Na_2HPO_4, then diluted in PBSi, frozen, and titrated in an identical manner. Virus samples were also mixed with ice-cold HDX-MS Quench buffer (150mM tris(2-carboxyethyl) phosphine-HCl, 2M urea, 0.1% formic acid final concentrations), vortexed briefly to mix, and incubated on ice for 2 min. Samples were then diluted 1/100 in PBSi for freezing prior to titration. Dotted line indicates detection limit of plaque assay, open symbols indicate data points below this limit. Data analyzed by One-Way ANOVA (ns, no significant difference, ** $p < 0.01$), n = 3 individual samples per treatment group.

Supplementary Table S1 – Statistical results from Ordinary Two-Way ANOVA with Tukey's Multiple Comparisons Test and a single pooled variance. Values here are related to Figure 2D, showing D_2O incorporation into individual peptides from IAV protein Hemagglutinin (HA) with time. The summary of significance (Sig.) and adjusted P-values for each individual comparison are reported. Values indicate the statistical comparison of means from triplicate samples exposed to pH 7 or pH 4 (variable 1) for 2 different time periods (variable 2). Statistical comparisons are grouped according to the incubation time of samples in deuterium oxide (D_2O, 3 different incubation times utilized for analysis of structural changes).

TWO-WAY ANOVA RESULTS	HA1 Peptide 51-58		HA1 Peptide 68-79		HA1 Peptide 81-92		HA1 Peptide 89-95		HA1 Peptide 113-117		HA1 Peptide 317-328		HA2 Peptide 521-532	
with Tukey's Multiple Comparisons Test	*Sig.*	*P- Value*	*Sig.*	*P-Value*	*Sig.*	*P- Value*	*Sig.*	*P- Value*	*Sig.*	*P- Value*	*Sig.*	*P- Value*	*Sig.*	*P-Value*
**D_2O - 30 sec**														
pH 7 - 10 sec vs. pH 7 - 30 sec	ns	0.6465	ns	0.8535	ns	0.2907	ns	0.9956	ns	>0.9999	ns	0.485	ns	0.5725
pH 7 - 10 sec vs. pH 4 - 10 sec	ns	0.9862	*	0.0115	*	0.0244	ns	0.556	****	<0.0001	****	<0.0001	****	<0.0001
pH 7 - 30 sec vs. pH 4 - 30 sec	ns	0.0884	ns	0.1024	****	<0.0001	ns	0.5173	****	<0.0001	****	<0.0001	****	<0.0001
pH 4 - 10 sec vs. pH 4 - 30 sec	ns	0.7697	ns	0.2798	*	0.0111	ns	0.9909	ns	0.9732	ns	0.8309	ns	0.7823
**D_2O - 300 sec**														
pH 7 - 10 sec vs. pH 7 - 30 sec	ns	0.9696	ns	0.2266	ns	0.9963	ns	0.9021	ns	0.9994	ns	0.9997	ns	0.9919
pH 7 - 10 sec vs. pH 4 - 10 sec	****	<0.0001	ns	0.096	ns	0.0947	ns	0.0555	****	<0.0001	****	<0.0001	****	<0.0001
pH 7 - 30 sec vs. pH 4 - 30 sec	****	<0.0001	ns	0.1763	****	<0.0001	**	0.0075	****	<0.0001	****	<0.0001	****	<0.0001
pH 4 - 10 sec vs. pH 4 - 30 sec	ns	0.5621	ns	0.078	*	0.0121	ns	0.9932	ns	0.56	ns	0.9988	ns	0.9998
**D_2O – 3,600 sec**														
pH 7 - 10 sec vs. pH 7 - 30 sec	ns	0.9994	ns	0.2978	ns	0.9689	ns	0.9873	ns	0.549	ns	0.9777	ns	0.9025
pH 7 - 10 sec vs. pH 4 - 10 sec	****	<0.0001	ns	0.9997	ns	0.9999	****	<0.0001	****	<0.0001	****	<0.0001	****	<0.0001
pH 7 - 30 sec vs. pH 4 - 30 sec	****	<0.0001	ns	0.0791	*	0.0446	****	<0.0001	****	<0.0001	****	<0.0001	****	<0.0001
pH 4 - 10 sec vs. pH 4 - 30 sec	ns	0.9766	ns	0.8682	ns	0.1272	ns	0.961	ns	0.9965	ns	0.9929	ns	0.9997

Supplementary Table S2 – Statistical results from Ordinary Two-Way ANOVA with Tukey's Multiple Comparisons Test and a single pooled variance. Values here are related to Figure 6C, showing D_2O incorporation into individual peptides from IAV protein Matrix 1 (M1) with time. The summary of significance (Sig.) and adjusted P-values for each individual comparison are reported. Values indicate the statistical comparison of means from triplicate samples exposed to pH 7 or pH 4 (variable 1) for 2 different time periods (variable 2). Statistical comparisons are grouped according to the incubation time of samples in deuterium oxide (D_2O, 3 different incubation times utilized for analysis of structural changes).

**TWO-WAY ANOVA RESULTS**	**M1 Peptide 33-41**		**M1 Peptide 100-114**		**M1 Peptide 128-141**		**M1 Peptide 131-141**		**M1 Peptide 168-181**		**M1 Peptide 182-197**		**M1 Peptide 216-229**		**M1 Peptide 234-239**		**M1 Peptide 240-252**	
with Tukey's Multiple Comparisons Test	*Sig.*	*P-Value*	*Sig.*	*P-Value*	*Sig.*	*P-Value*	*Sig.*	*P-Value*	*Sig.*	*P-Value*	*Sig.*	*P-Value*	*Sig.*	*P-Value*	*Sig.*	*P-Value*	*Sig.*	*P-Value*
**D_2O - 30 sec**																		
pH 7 - 10 sec vs. pH 7 - 30 sec	ns	0.3004	ns	0.8953	ns	0.1325	ns	0.4019	ns	0.8556	ns	0.6898	ns	0.7543	ns	0.6888	ns	0.5155
pH 7 - 10 sec vs. pH 4 - 10 sec	ns	0.152	ns	0.9877	*	0.0395	ns	0.0994	ns	0.8438	ns	0.5493	ns	0.8449	***	0.0002	ns	0.0521
pH 7 - 30 sec vs. pH 4 - 30 sec	ns	0.8994	ns	0.1723	**	0.0095	*	0.0299	ns	0.2553	****	<0.0001	****	<0.0001	****	<0.0001	****	<0.0001
pH 4 - 10 sec vs. pH 4 - 30 sec	ns	0.9147	ns	0.2863	*	0.0371	ns	0.1629	ns	0.9921	****	<0.0001	***	0.0001	****	<0.0001	****	<0.0001
**D_2O - 300 sec**																		
pH 7 - 10 sec vs. pH 7 - 30 sec	ns	0.9995	ns	0.7973	ns	0.941	ns	0.9974	ns	0.9251	ns	>0.9999	ns	0.4184	ns	>0.9999	ns	0.8783
pH 7 - 10 sec vs. pH 4 - 10 sec	ns	0.9861	ns	0.9355	ns	0.7857	ns	0.6237	ns	0.3704	***	0.0005	*	0.024	****	<0.0001	**	0.0098
pH 7 - 30 sec vs. pH 4 - 30 sec	ns	0.9204	ns	0.9712	****	<0.0001	***	0.0004	ns	0.1009	****	<0.0001	**	0.0033	****	<0.0001	****	<0.0001
pH 4 - 10 sec vs. pH 4 - 30 sec	ns	0.9773	ns	0.8925	**	0.0018	*	0.0112	ns	0.5214	****	<0.0001	*	0.0259	****	<0.0001	**	0.0096
**D_2O – 3,600 sec**																		
pH 7 - 10 sec vs. pH 7 - 30 sec	ns	0.75	ns	0.0728	ns	0.8682	ns	0.5594	ns	>0.9999	ns	0.9931	ns	0.8614	ns	0.9989	ns	0.9997
pH 7 - 10 sec vs. pH 4 - 10 sec	ns	0.8569	ns	0.7913	**	0.0061	**	0.0015	**	0.0037	****	<0.0001	ns	0.2344	*	0.0106	ns	0.5318
pH 7 - 30 sec vs. pH 4 - 30 sec	ns	0.2391	ns	0.8292	***	0.0004	***	0.0005	****	<0.0001	****	<0.0001	***	0.0003	****	<0.0001	**	0.0078
pH 4 - 10 sec vs. pH 4 - 30 sec	ns	0.4029	**	0.0098	ns	0.2498	ns	0.3105	***	0.0002	****	<0.0001	ns	0.1374	****	<0.0001	ns	0.1774

Supplementary Tables S3, S4, S5 – External Excel tables listing HDX-MS raw data including the list of peptide analyzed, deuteration levels and differences in deuteration levels for each timepoint. One table is included for each protein studied (HA; NP; M1). Accessible online.

```
                                1                                                                  Peptide 1                            Peptide 2 & 3  100
1. H1N1 A/PR8             ...  MKANLL-----VLLC----ALAAADADTICIGYHANNSTDTVDTVLEKNVTVTHSVNLLEDSHNGKLCRLKGIAPLQLGKCNIAGWLLGNPECDPLLPVR
2. H1N1 MA(USA) 2023      ...  MKATLV-----VMLY----TFTTANADTLCIGYHANNSTDTVDTVLEKNVTVTHSVNLLEDKHNGKLCKLRGVAPLHLGQCNIAGWILGNPECESLSTAR
3. H1N1 Alaska 2023       ...  MKATLV-----VMLY----TFTTANADTLCIGYHANNSTDTVDTVLEKNVTVTHSVNLLEDKHNGKLCKLRGVAPLHLGQCNIAGWILGNPECESLSTAR
4. H1N1 Germany 2023      ...  MKATLV-----VMLY----TFTTANADTLCIGYHANNSTDTVDTVLEKNVTVTHSVNLLEDKHNGKLCKLRGIAPLHLGQCNIAGWILGNPECESLSTAR
5. H1N1 UK 2023           ...  MKATLV-----VMLY----TFTTANADTLCIGYHANNSTDTVDTVLEKNVTVTHSVNLLEDKHNGKLCKLRGVAPLHLGQCNIAGWILGNPECESLSTAR
6. pdmH1N1 A/Cali/07/... MKATLV-----VLLY----TFATANADTLCIGYHANNSTDTVDTVLEKNVTVTHSVNLLEDKHNGKLCKLRGVAPLHLGKCNIAGWILGNPECESLSTAS
7. H3N2 Germany 2023      ...  MKAIIALSNILCLVFAQKIPGNDNSTATLCLGHHAVPNGTIVKTITNDRIEVTNATELVQNSSIGKICNSPH-QILDGGNCTLIDALLGDPQCDGFQN-K
8. H3N2 WI(USA) 2023      ...  MKAIIALSNILCLVFAQKIPGNDNSTATLCLGHHAVPNGTIVKTITNDRIEVTNATELVQNSSIGKICNSPH-QILDGGNCTLIDALLGDPQCDGFQN-K
9. H3N2 Japan 2023        ...  MKAIIALSNILCLVFAQKIPGNDNSTATLCLGHHAVPNGTIVKTITNDRIEVTNATELVQNSSIGKICNSPH-QILDGGNCTLIDALLGDPQCDGFQN-K

                                101                     Peptide 4                                                                   200
1. H1N1 A/PR8             ...  SWSYIVETPNSENGICYPGDFIDYEELREQLSSVSSFERFEIFPKESSWPNHNT-NGVTAACSHEGKSSFYRNLLWLTEKEGSYPKLKNSYVNKKGKEVL
2. H1N1 MA(USA) 2023      ...  SWSYIVETSNSDNGTCYPGDFINYEELREQLSSVSSFERFEIFPKTSSWPNHDSDNGVTAACSHAGARSFYKNLIWLVKKGKSYPKINQTYINDKGKEVL
3. H1N1 Alaska 2023       ...  SWSYIVETSNSDNGTCYPGDFINYEELREQLSSVSSFERFEIFPKTSSWPNHDSDNGVTAACSHAGARSFYKNLIWLVKKGKSYPKINQTYINDKGKEVL
4. H1N1 Germany 2023      ...  SWSYIVETPNPDNGTCYPGNFINYEELREQLSSVSSFERFEIFPKTSSWPNHDSDNGVTAACPHAGAKSFYKNLIWLVKKGKSYPKINQTYINDKGKEVL
5. H1N1 UK 2023           ...  SWSYIVETSNSDNGTCYPGDFINYEELREQLSSVSSFERFEIFPKTSSWPNHDSDNGVTAACSHAGARSFYKNLIWLVKKGKSYPKINQTYINDKGKEVL
6. pdmH1N1 A/Cali/07/. ..  SWSYIVETPSSDNGTCYPGDFIDYEELREQLSSVSSFERFEIFPKTSSWPNHDSNKGVTAACPHAGAKSFYKNLIWLVKKGNSYPKLSKSYINDKGKEVL
7. H3N2 Germany 2023      ...  EWDLFVER-NRANSSCYPYDVPDYASLRSLVASSGTL---EFKDESFNWTGV-KQNGTSSACKRGSSSSFFSRLNWLTSLNNIYPAQNVTMPNKEQFDKL
8. H3N2 WI(USA) 2023      ...  EWDLFVER-SRANSSCYPYDVPDYASLRSLVASSGTL---EFKNESFNWTGV-KQNGTSSACKRGSSSSFFSRLNWLTSLNNIYPAQNVTMPNKEQFDKL
9. H3N2 Japan 2023        ...  EWDLFVER-SRANSSCYPYDVPDYASLRSLVASSGTL---EFKNESFNWTGV-KQNGTSSACKRGSSSSFFSRLNWLTSLNNIYPAQNVTMPNKEQFDKL

                                201                                                                                                 300
1. H1N1 A/PR8             ...  VLWGIHHPPNSKEQQNLYQNENAYVSVVTSNYNRRFTPEIAERPKVRDQAGRMNYYWTLLKPGDTIIFEANGNLIAPMYAFALSRGFGSGIITSNASMHE
2. H1N1 MA(USA) 2023      ...  VLWGIHHPPTITDQESLYQNADAYVFVGTSRYSKKFKPEIATRPKVRDQAGRMNYYWTLVEPGDKITFEATGNLVAPRYAFTMEKEAGSGIIISDTPVHD
3. H1N1 Alaska 2023       ...  VLWGIHHPPTITDQESLYQNADAYVFVGTSRYSKKFKPEIAARPKVRDQAGRMNYYWTLVEPGDKITFEATGNLVAPRYAFTMEKEAGSGIIISDTPVHD
4. H1N1 Germany 2023      ...  VLWGIHHPPTITDQESLYQNADAYVFVGTSRYSKKFKPEIAARPKVRDQAGRMNYYWTLVDPGDKITFEATGNLVAPRYAFTMEKDAGSGIIISDTPVQD
5. H1N1 UK 2023           ...  VLWGIHHPPTITDQESLYQNADAYVFVGTSRYSKKFKPEIATRPKVRDQAGRMNYYWTLVEPGDKITFEATGNLVAPRYAFTMEKEAGSGIIISDTPVHD
6. pdmH1N1 A/Cali/07/. ..  VLWGIHHPSTSADQQSLYQNADAYVFVGSSRYSKKFKPEIATRPKVRXXEGRMNYYWTLVEPGDKITFEATGNLVVPRYAFAMERNAGSGIIISDTPVHD
7. H3N2 Germany 2023      ...  YIWGVHHPDTDKNQFSLFAQSSGRITVSTKRSQQAVIPNIGSRPRVRDIPSRISIYWTIVKPGDILLINSTGNLIAPRGYFKIRSG-KSSIMRSDAPIGK
8. H3N2 WI(USA) 2023      ...  YIWGVHHPDTDKNQFSLFAQSSGRITVSTKRSQQAVIPNIGSRPRVRDIPSRISIYWTIVKPGDILLINSTGNLIAPRGYFKIRSG-KSSIMRSDAPIGK
9. H3N2 Japan 2023        ...  YIWGVHHPDTDKNQFSLFAQSSGRITVSTKRSQQAVIPNIGSRPRVRDIPSRISIYWTIVKPGDILLINSTGNLIAPRGYFKIRSG-KSSIMRSDAPIGK

                                301                            Peptide 5                                                            400
1. H1N1 A/PR8             ...  CNTKCQTPLGAINSSLPYQNIHPVTIGECPKYVRSAKLRMVTGLRNIPSIQSRGLFGAIAGFIEGGWTGMIDGWYGYHHQNEQGSGYAADQKSTQNAING
2. H1N1 MA(USA) 2023      ...  CNATCQTPEGAINTSLPFQNVHPITIGKCPKYVRSTKLRLATGLRNVPSIQSRGLFGAIAGFIEGGWTGMVDGWYGYHHQNDQGSGYAADLKSTQNAIDK
3. H1N1 Alaska 2023       ...  CNATCQTPEGAINTSLPFQNVHPITIGKCPKYVRSTKLRLATGLRNVPSIQSRGLFGAIAGFIEGGWTGMVDGWYGYHHQNDQGSGYAADLKSTQNAIDK
4. H1N1 Germany 2023      ...  CNTTCQTPEGAINTSLPFQNVHPITIGKCPKYVRSTKLRLATGLRNIPSIQSRGLFGAIAGFIEGGWTGMVDGWYGYHHQNEQGSGYAADLKSTQNAIDK
5. H1N1 UK 2023           ...  CNATCQTPEGAINTSLPFQNVHPITIGKCPKYVRSTKLRLATGLRNVPSIQSRGLFGAIAGFIEGGWTGMVDGWYGYHHQNDQGSGYAADLKSTQNAIDK
6. pdmH1N1 A/Cali/07/. ..  CNTTCQTPKGAINTSLPFQNIHPITIGKCPKYVKSTKLRLATGLRNIPSIQSRGLFGAIAGFIEGGWTGMVDGWYGYHHQNEQGSGYAADLKSTQNAIDE
7. H3N2 Germany 2023      ...  CKSECITPNGSIPNDKPFQNVNRITYGACPRYVKQSTLKLATGMRNVPEKQTRGIFGAIAGFIENGWEGMVDGWYGFRHQNSEGRGQAADLKSTQAAIDQ
8. H3N2 WI(USA) 2023      ...  CKSECITPNGSIPNDKPFQNVNRITYGACPRYIKQSTLKLATGMRNVPEKQTRGIFGAIAGFIENGWEGMVDGWYGFRHQNSEGRGQAADLKSTQAAIDQ
9. H3N2 Japan 2023        ...  CKSECITPNGSIPNDKPFQNVNRITYGACPRYVKQSTLKLATGMRNVPEKQTRGIFGAIAGFIENGWEGMVDGWYGFRHQNSEGRGQAADLKSTQAAIDQ
```

```
                               401      β-loop residues                                                            500
1. H1N1 A/PR8           ... ITNKVNTVIEKMNIQFTAVGKEFNKLEKRMENLNKKVDDGFLDIWTYNAELLVLLENERTLDFHDSNVKNLYEKVKSQLKNNAKEIGNGCFEFYHKCDNE
2. H1N1 MA(USA) 2023 ... ITNKVNSVIEKMNTQFTAVGKEFNHLEKRIENLNKKVDDGFLDVWTYNAELLVLLENERTLDYHDSNVKNLYEKVRHQLKNNAKEIGNGCFEFYHKCDNT
3. H1N1 Alaska 2023  ... ITNKVNSVIEKMNTQFTAVGKEFNHLEKRIENLNKKVDDGFLDVWTYNAELLVLLENERTLDYHDSNVKNLYEKVRHQLKNNAKEIGNGCFEFYHKCDNT
4. H1N1 Germany 2023 ... ITNKVNSVIEKMNTQFTAVGKEFNHLEKRIENLNKKVDDGFLDIWTYNAELLVLLENERTLDYHDSNVKNLYEKVRNQLKNNAKEIGNGCFEFFHKCDNT
5. H1N1 UK 2023      ... ITNKVNSVIEKMNTQFTAVGKEFNHLEKRIENLNKKVDDGFLDVWTYNAELLVLLENERTLDYHDSNVKNLYEKVRHQLKNNAKEIGNGCFEFYHKCDNT
6. pdmH1N1 A/Cali/07/... ITNKVNSVIEKMNTQFTAVGKEFNHLEKRIENLNKKVDDGFLDIWTYNAELLVLLENERTLDYHDSNVKNLYEKVRSQLKNNAKEIGNGCFEFYHKCDNT
7. H3N2 Germany 2023 ... ISGKLDRLIGKTNEKFHQIEKEFSEVEGRVQDLEKYVEDTKIDLWSYNAELLVALENQHTIDLTDSEMNKLFEKTKKQLRENAEDMGNGCFKIYHKCDNA
8. H3N2 WI(USA) 2023 ... ISGKLNRLIGKTNEKFHQIEKEFSEVEGRVQDLEKYVEDTKIDLWSYNAELLVALENQHTIDLTDSEMNKLFEKTKKQLRENAEDMGNGCFKIYHKCDNA
9. H3N2 Japan 2023   ... ISGKLNRLIGKTNEKFHQIEKEFSEVEGRVQDLEKYVEDTKIDLWSYNAELLVALENQHTIDLTDSEMNKLFEKTKKQLRENAEDMGNGCFKIYHKCDNA

                               501                              Peptide 6                         575
1. H1N1 A/PR8           ... CMESVRNGTYDYPKYSEESKLNREKVDGVKLESMGIYQILAIYSTVASSLVLLVSLGAISFWMCSNGSLQCRICI
2. H1N1 MA(USA) 2023 ... CMESVKNGTYDYPKYSEEAKLNREKIDGVKLDSTRIYQILAIYSTVASSLVLVVSLGAISFWMCSNGSLQCRICI
3. H1N1 Alaska 2023  ... CMESVKNGTYDYPKYSEEAKLNREKIDGVKLDSTRIYQILAIYSTVASSLVLVVSLGAISFWMCSNGSLQCRICI
4. H1N1 Germany 2023 ... CMESVKNGTYDYPKYSEEAKLNREKIDGVKLDSTRIYQTLAIYSTVASSLVLVVSLGAISFWMCSNGSLQCRICI
5. H1N1 UK 2023      ... CMESVKNGTYDYPKYSEEAKLNREKIDGVKLDSTRIYQILAIYSTVASSLVLVVSLGAISFWMCSNGSLQCRICI
6. pdmH1N1 A/Cali/07/... CMESVKNGTYDYPKYSEEAKLNREEIDGVKLESTRIYQILAIYSTVASSLVLVVSLGAISFWMCSNGSLQCRICI
7. H3N2 Germany 2023 ... CIGSIRNETYDHNVYRDEALNNRFQIKGVELKSGYKDWILWI-SFAMSCFLLCIALLGFIMWACQKGNIRCNICI
8. H3N2 WI(USA) 2023 ... CIGSIRNETYDHNVYRDEALNNRFQIKGVELKSGYKDWILWI-SFAMSCFLLCIALLGFIMWACQKGNIRCNICI
9. H3N2 Japan 2023   ... CIGSIRNETYDHNVYRDEALNNRFQIKGVELKSGYKDWILWI-SFAMSCFLLCIALLGFIMWACQKGNIRCNICI
```

Supplementary Figure S8 – Multi-sequence alignment of A/PR8 haemagglutinin (HA) to the HA sequences of recent circulating human IAV isolates. Seasonal subtypes H1N1 and H3N2 isolated in 2023 from diverse geographical locations are included in this alignment, along with an isolate of the 2009 swine flu pandemic (pdmH1N1). All sequences were obtained from the Influenza Virus Database (NCBI), with multi-sequence alignment and annotation performed using the Clustal Omega Multi sequence alignment tool with a user interface from Benchling. Mismatches between A/PR8 and the circulating human sequences are highlighted orange. Peptides 1-6 identified by HDX-MS in main text Figure 2D are highlighted blue, along with the residues associated with the HA2 β-loop. The HA fusion peptide is underlined. Average sequence identity for A/PR8 compared to all H1N1 isolates here was 79%, whilst for H3N2 the sequence identity with A/PR8 was approximately 40%. Accession numbers for sequences included here are: H1N1 A/PR8/34 [P03452], H1N1 Massachusetts USA 2023 [WHO41931], H1N1 Alaska 2023 [WEI46805], H1N1 Germany 2023 [WGD05227], H1N1 UK 2023 [WEI47076], pdmH1N1 A/Cali/07/2009 [ACP44189.1], H3N2 Germany 2023 [WGD05425], H3N2 Wisconsin USA 2023 [WHO53914], and H3N2 Japan 2023 [WGD05230].

```
                                 1                                                                             Peptide 1                       Peptide 2 & 3 100
H1N1 A/PR8                   ... -MKA--NLLVLLCALAA-------ADADTICIGYHANNSTDTVDTVLEKNVTVTHSVNLLEDSHNGKLCRLKGIAPLQLGKCNIAGWLLGNPECDPLLPV
H1N1 2022 (Swine, US)...        -MKA--ILLVLLHTLA-------TATADTICIGYHANNSTDTVDTVLEKNVTVTHSVNLLEDKHNGKLCKLGGKSPLYLGKCNIAGWLLGNPECESILTV
H1N2 2022 (Swine, UK)...        -MKA--KLIVLFCALS-------ATYADTICIGYHANNSTDTVDTILEKNVTVTHSVNLLENSHNGKLCRMKGIAPLQLGKCSIAGWILGNPECESSFSR
H3N2 2022 (Swine, US)..         -MKTIIAFSCILCLIIAQKLPGSDNSMATLCLGHHAVPNGTLVKTITDDQIEVTNATELVQSSSTGRICN-SPHQILDGKNCTLIDALLGDPHCDDFQN-
H3N2 2023 (Swine, US)...        -MKTIIALSHILCLVFAQKLHGNDNNMATLCLGHHAVPNGTIVKTITNDQVEVTNATELVQSSSKGEICN-SPYQILDGENCTLIDALLGDPQCDGFQN-
H3N8 2022 (Av, Canada...        -MKTVIALSYIFCLTFGQDLSGNDNSTATLCLGHHAVPNGTIVKTITDDQIEVTNATELVQSSSTGKICN-NPHRILDGRDCTLVDALLGDPHCDVFQD-
H3N8 2022 (Av, China)...        -MKTIIALNYILCLAFGQNLPGKDSSTATLCLGHHSVPNGTIVKTITDDQIEVTNATELVQNSSTGKICN-SPHKVLDGRDCTLIDAVLGDPHCDVFQD-
H5N1 2015 (Av, Viet)  ...       --ME--KIVLLFATIS-------LVKSDHICIGYHANNSTEQVDTIMEKNVTVTHAQDILEKTHNGKLCDLNGVKPLILKDCSVAGWLLGNPLCDEFTNV
H5N1 2022 (Av, Japan)...        --ME-NIVLLLAIVS--------LVKSDQICIGYHANNSTEQVDTIMEKNVTVTHAQDILEKTHNGKLCDLNGVKPLILKDCSVAGWLLGNPMCDEFIRV
H7N9 2017 (Av, US)    ...       -MNT-QILALIACMLIGA-------KGDKICLGHHAVANGTKVNTLTERGIEVVNATETVETANIKKICT-QGRRPTDLGQCGLLGTLIGPPQCDQFLE-
H9N2 2015 (Av, Japan)...        METV-SLITILLVAT--------VSNADKICIGYQSTNSTETVDTLTENNVPVTHAKELLHTEHNGMLCATSLGQPLILDTCTIEGLIYGNPSCDLSLEG

                                 101                 Peptide 4                                                                   200
H1N1 A/PR8                   ... RSWSYIVETPNSENGICYPGDFIDYEELREQLSSVSSFERFEIFPKESSWPNHNT-NGVTAACSHEGKSSFYRNLLWLTEKE--GSYPKLKNSYVNKKGK
H1N1 2022 (Swine, US)...        SSWSYIVETSNSDNGTCYPGSFTNYEELREQLSSVSSFEKFEIFPIESSWSNYK--TGETASCPYAGKSSFYRNLMWLVKKA--DSYPRLNISYVNNKGK
H1N2 2022 (Swine, UK)...        KSWSYIAETPNPENGICYPGYFSDYEELREQLSSVSSVERFEMFPKESSWPKHNVTRGKTMSCSHNGKSSFYKNLLWLTEKD--GLYPNISNHYVNNKEK
H3N2 2022 (Swine, US)...        KEWDLFVERSTA-YSSCYPYYVPDHATLRSLVASFGNLKFTQ---EIFNWTGV-TQDGSSYSCRRGSVNGFFSRLNWLYHLD--HKYPEQNVTMPNNDKF
H3N2 2023 (Swine, US)...        SKWDLFVERSKA-HSNCYPYDVPDYASLRSLIASSGTLEFTN---ESFNWTGV-TQDGTSSSCKRRSNNSFFSRLNWLTHLN--YKYPALEVTMPNNEQF
H3N8 2022 (Av, Canada...        ETWDLFVERSNA-FSNCYPYDVPDYASLRSLVASSGTLEFIT---EGFTWTGV-TQNGGSNACKRGPASGFFSRLNWLTKSG--NAYPVLNVTMPNNDNF
H3N8 2022 (Av, China)...        EKWDLFVERSSA-FSNCYPYDVPDYASLRSLIASSGTLDFIT---ESFTWAGV-SQNGGSGACKRGPANGFFSRLNWLTKSG--SSYPLLNVTMPNNYNF
H5N1 2015 (Av, Viet)  ...       PEWSYIVEKANPANDLCYPGNFNDYEELKHLLSRINHFEKIQIIPKD-SWSDHEASLGVSAACSYQGNSSFFRNVVWLIKKN--NAYPTIKKGYNNTNRE
H5N1 2022 (Av, Japan)...        PEWSYIVEKANPTNDLCYPGSLNDYEELKHLLSRINHFEKILIIPKN-SWPNHETSLGVSAACPYQGAPSFFRNVVWLIKKN--DAYPTIKISYNNTNRE
H7N9 2017 (Av, US)    ...       FDADLITERREG-TDVCYPGKFTNEESLRQILRGSGGIDKES---MGFTYSGI-RTNGATSACRRS-GSSFYAEMKWLLSNSNNAAFPQMTKSYRNPRSK
H9N2 2015 (Av, Japan)...        REWSYIVERPSAVNGLCYPGNVENLEELRSLFSSARSYQRIQIFPDT-IWNVSY--DGTSTAC----SGSFYKSMRWLTRKN--GDYPIQDAQYTNNQGK

                                 201                                                                                                 300
H1N1 A/PR8                   . ..EVLVLWGIHHPPNSKEQQNLYQNENAYVSVVTSNYNRRFTPEIAERPKVRDQAGRMNYYWTLLKPGDTIIFEANGNLIAPMYAFALSRGFGSGIITSNAS
H1N1 2022 (Swine, US). ..        EVLVLWGIHHPPTGNDQQWLYQNANASVFVGTSTYSQKFKPEIATRPKVRGQAGRMNYHWTLVEPGDTITFEATGNLVVPRYAFAMNRGSGSGIIVSDAP
H1N2 2022 (Swine, UK). ..        EVLVIWGVHNPSNMEDQRAIYRKENAYVSVVSSHYNRRFTPEIEKRPKIRNQEGRINYYWTLLEPGETIIFKANGNFIAPRYAFALSRGHRSGIIISNAS
H3N2 2022 (Swine, US). ..        DKLYIWGVHHPGTDKDQASLYVQASGRVIVSTRRSQQTVIPNIGSRPWVRGVSSIISIYWTIVKPGDILLINSTGNLIAPRGYFKI-QSGKSSIMRSDAH
H3N2 2023 (Swine, US). ..        DKLYIWGVHHPATDKDQIYLYAQAEGKIIVSTKRSQQAVIPNIGSRPRIRDIPSRISIYWTIVRPGDTLLINSTGNLIAPRGYFKI-RSGKSSIMRSDAP
H3N8 2022 (Av, Canada. ..        DKLYIWGVHHPSTNQEQTNLYVQASGRVTVSTRRSQQTIIPNIGSRPWVRGQSGRISIYWTIVKPGDVLVINSNGNLIAPRGYFKM-RTGKSSIMKSDAP
H3N8 2022 (Av, China). ..        DKLYIWGVHHPSTNQEQTNLYVQASGRVTVSTRRSQQTIVPNIGSRPWVRGQSGRISIYWTIVKPGDVLVINSNGNLIAPRGFFKI-RTGRSSIMRSDAP
H5N1 2015 (Av, Viet)  ...       DLLILWGIHHPNDEAEQTRLYQNPTTYISIGTSTLNQRLVPKIATRSKINGQSGRIDFFWTILKPNDTIHFESNGNFIAPEYAYKIVKKGDSTIMRSEVK
H5N1 2022 (Av, Japan). ..        DLLILWGIHHSNNAEEQTDLYKNPTTYISVGTSTLNQRLVPKIATRSQVNGQRGRMDFFWTILKPDDAIHFESNGNFIAPEYAYKIVKKGDSTIMKSGVE
H7N9 2017 (Av, US)    ...       PALIIWGVHHSGSAAEQTKLYGSGNKLITVGSSKYQQSFTPSPGARPQVNGQSGRIDFHWLLLDPNDTVTFTFNGAFIAPDRASFL-RG-ESLGVQSDVP
H9N2 2015 (Av, Japan). ..        NILFMWGINHPPTDTTQRDLYTRIDTTTSVATEGINRVFKPLIGPRPLVNG-LGRIDYYWSVLKPGQTLRIKSDGNLIAPWFGHILSGESHGRILKTDLK
```

```
                                   Peptide 5
                         301                                                                                          400
H1N1 A/PR8              ... M-HECNTKCQTPLGAINSSLPYQNIHPVTIGECPKYVRSAKLRMVTGLRNIPSI----------QSRGLFGAIAGFIEGGWTGMIDGWYGYHHQNEQGSG
H1N1 2022 (Swine, US)...    I-HDCNTTCQTPKGALNTSLPFQNVHPVTIGECPKYVKSRRLKMATGLRNIPSI----------QSRGLFGAIAGFIEGGWTGMVDGWYGYHHQNDQGSG
H1N2 2022 (Swine, UK)...    M-DECDTECQTPQGAINNSLPFQNVHPVAIGECPKYIKSTRLRMVTGLRNIPSI----------QSRGLFGAIAGFIEGGWTGMIDGWYGYHHRNEQGSG
H3N2 2022 (Swine, US)...    I-DECNSECITPNGSISNDKPFQNVNKITYGACPRYVKQNTLKLATGMRNIPEK----------QTRGIFGAIAGFIENGWEGMVDGWYGFRHQNSEGTG
H3N2 2023 (Swine, US)...    I-GKCNSACITPNGSIPNDKPFQNVNRITYGACPRYVKQNTLKLATGMRNVPEK----------QTRGIFGAIAGFIENGWEGMVDGWYGFRHQNSEGRG
H3N8 2022 (Av, Canada...    I-DTCISECITPNGSIPNDKPFQNVNKITYGACPKYVKQNTLKLATGMRNVPEK----------QTRGLFGAKAGFIENGWEGMIDGWYGFRHQNSEGTG
H3N8 2022 (Av, China)...    I-ETCISECITPNGSIPNDKPFQNVNKITYGACPKYVKQNTLKLATGMRNVPEK----------QTRGLFGAIAGFIENGWEGMIDGWYGFRHQNSEGTG
H5N1 2015 (Av, Viet) ...    Y-GNCNTRCQTPIGAINSSMPFHNIHPLTIGECPKYVKSNKLVLATGLRNSPQR-------ERRRKRGLFGAIAGFIEGGWQGMVDGWYGYHHSNEQGSG
H5N1 2022 (Av, Japan)...    Y-GHCNTKCQTPVGAINSSMPFHNIHPLTIGECPKYVKSNKLVLATGLRNSPLR-------ERRRKRGLFGAIAGFIEGGWQGMVDGWYGYHHSNEQGSG
H7N9 2017 (Av, US)    ...   LDSSCEGDCFHNGGTIVSSLPFQNINPRTVGKCPRYVKQTSLLLATGMRNVPENPKTDRKSRHRRIRGLFGAIAGFIENGWEGLIDGWYGFRHQNAQGEG
H9N2 2015 (Av, Japan)...    R-GSCTVQCQTEKGGLNSTLPFQNVSKYAFGNCSKYIGIKSLKLAVGLRNVPSR----------SSRGLFGAIAGFIEGGWSGLVAGWYGFQHSNDQGVG
```

```
                                  β-loop residues
                         401                                                                                          500
H1N1 A/PR8             . . YAADQKSTQNAINGITNKVNTVIEKMNIQFTAVGKEFNKLEKRMENLNKKVDDGFLDIWTYNAELLVLLENERTLDFHDSNVKNLYEKVKSQLKNNAKEI
H1N1 2022 (Swine, US). . YAADQKSTQRAIDGITNKVNSVIEKMNTQFTAVGKEFNNLERRIENLNRKVDDGFLDVWTYNAELLVLLENERTLDFHDSNVKNLYEKVRNQLRNNAKEI
H1N2 2022 (Swine, UK). . FAADQESTQNAINGVTNKVNSIIEKMNTQFTAAGKEFNRLEKRMENLNKKVDDGLLDVWTYNAELLVLLENERTLDFHDSNVKNLYERVKSQLKNNAKEI
H3N2 2022 (Swine, US). . QAADLESTQSAINQITGKLNRVIKKTNEKFHQIEKEFSEVEGRIQDLEKYVEDTKIDLWSYNAELLVALENQHTIDLTDSEMNKLFERTRRQLRENAEDM
H3N2 2023 (Swine, US). . QAADLKSTQAAIDQINGKLNRLIGKTNEKFHQIEKEFSEVEGRIQDLEKYVEDTKIDLWSYNAELLVALENQHTIDLTDSEMSKLFEKTKKQLRENAEDM
H3N8 2022 (Av, Canada. . QAADLKSTQAAIDQINGKLNRVIEKTNEKFHQIEKEFSEVEGRIQDLEKYVEDTKIDLWSYNAELLVALENQHTIDLTDSEMNKLFEKTRRQLRENAEDM
H3N8 2022 (Av, China). . QAADLKSTQAAIDQINGKLNRVIEKTNEKFHQIEKEFSEVEGRIQDLEKYVEDTKVDLWSYNAELLVALENQHTIDLTDSEMNKLFEKTRRQLRENAEDM
H5N1 2015 (Av, Viet)  ... YAADRESTQKAIDGVTNKVNSIIDKMNTQFEAVGREFNNLERRIENLNKKMEDGFLDVWTYNAELLVLMENERTLDFHDSNVKNLYDKVRLQLKDNAKEL
H5N1 2022 (Av, Japan). . YAADKESTQKAIDGVTNKVNSIIDKMNTQFEAVGREFNNLERRIENLNKKMEDGFLDVWTYNAELLVLMENERTLDFHDSNVKNLYDKVRLQLRDNAKEL
H7N9 2017 (Av, US)    ... TAADYKSTQSAIDQITGKLNRLIDKTNQQFELIDNEFSEIEQQIGNVINWTRDSMTEVWSYNAELLVAMENQHTIDLADSEMNKLYERVRKQLRENAEED
H9N2 2015 (Av, Japan). . MAADRDSTQKAIDKITSKVNNIVDKMNKQYEIIDHEFSEVETRLNMINNKIDDQIQDIWAYNAELLVLLENQKTLDEHDANVNNLYNKVKRALGSNAVED
```

```
                                                        Peptide 6
                         501                                                                           589
H1N1 A/PR8             . . GNGCFEFYHKCDNECMESVRNGTYDYPKYSEESKLNREKVDGVKLESMGIYQILAIYSTVASSLVLLVSLGAISFWMCSNGSLQCRICI
H1N1 2022 (Swine, US). . GNGCFEFYHKCDNTCMESVKNGTYNYLKYSEESKLNREEIDGVKLDSTRVYQILAIYSTVASSLVLLVSLGALSFWMCSNGSLQCRICI
H1N2 2022 (Swine, UK). . GNGCFEFYHKCNNECMESVKNGTYDYPKYSEESNLNRRKIDGVKLESVDVYRILAIYSTVASSLVLLVSLGAISFWMCSNGSLQCRVCI
H3N2 2022 (Swine, US). . GNGCFKIYHKCDNACIGSIRNGTYDHDIYRNEALNNRFQVKGVQLKSGYKDW-ILWISFAISCFLLCVVLLGFIMWACQKGNIRCNICI
H3N2 2023 (Swine, US). . GNGCFKIYHKCDNACIGSIRNGTYDHDVYRDEALSNRFQIKGVELKSEYKDW-ILWISFAISCFLLCVALLGFIMWACQKGNIKCNICI
H3N8 2022 (Av, Canada. . GNGCFKIYHKCDNACIESIRNGTYDHDIYRDEALNNRFQIKGVELKSGYKDW-ILWISFAISCFLLCVVLLGFIMWACQRGNIRCNICI
H3N8 2022 (Av, China). . GNGCFKIYHKCDNACIESIRNGTYDHDIYRDEALNNRFQIRGVELKSGYKDW-ILWISFAISCFLLCVVLLGFIMWACQKGNIRCNICI
H5N1 2015 (Av, Viet)  ... GNGCFEFYHKCNNECMESVRNGTYDYPQYSEEARLKREEISGVKLESIGIYQILSIYSTVASSLVLAIMMAGLSLWMCSNGSLQCRICI
H5N1 2022 (Av, Japan). . GNGCFEFYHKCDNECMESVRNGTYDYPQYSEEARLKREEISGVKLESIGTYQILSIYSTAASSLALAIMMAGLSLWMCSNGSLQCRICI
H7N9 2017 (Av, US)    ... GTGCFEIFHKCDDQCMESIRNNTYDHTQYRAESLQNRIQIDPVKLSSGYKDI-ILWFSFGASCFLLLAIAMGLVFICIKNGNMRCTICI
H9N2 2015 (Av, Japan). . GKGCFELYHKCDDQCMETIRNGTYNRRKYQEESKLERQKIEGVKLESEGTYKILTIYSTVASSLVIAMGFAAFLFWAMSNGSCRCNICI
```

Supplementary Figure S9 – Multi-sequence alignment of A/PR8 haemagglutinin (HA) to the HA sequences of recent animal IAV isolates. Sequences of HA from H1N1, H1N2, and H3N2 IAV isolated from swine in 2022 and 2023, plus HA from avian (Av) strains H3N8, H5N1, H7N9, and H9N2 isolated between 2015 - 2022 were included in this alignment. All sequences were obtained from the Influenza Virus Database (NCBI), with multi-sequence alignment and annotation performed using the Clustal Omega Multi sequence alignment tool with a user interface from Benchling. Mismatches between A/PR8 and the circulating animal sequences are highlighted orange. Peptides 1-6 identified by HDX-MS in main text Figure 2D are highlighted blue, along with the residues associated with the HA2 β-loop. The HA fusion peptide is underlined. Average sequence identity for A/PR8 compared to animal H1N1 isolates here was 80%, whilst sequence identity was between 60 – 40% for other animal strains, with peptides of interest being less well-conserved here than in human isolates. Accession numbers for sequences included here are: H1N1

A/PR8/34 Human [P03452], H1N1 Swine US 2022 [UKU10399.1], H1N2 Swine UK 2022 [WGU23939.1], H3N2 Swine US 2022 [UMB33814.1], H3N2 Swine US 2023 [WIM36351], H3N8 Avian Canada 2022 [UZH94862], H3N8 Avian China 2022 [WBW07015], H5N1 Avian Vietnam 2015 [BAV31872], H5N1 Avian Japan 2022 [BDH01750.1], H7N9 Avian US 2017 [ARB51641], and H9N2 Avian Japan 2015 [BAW94758].
